# Benchmark for welding gun fault prediction with multivariate time series data

**DOI:** 10.1038/s41597-024-02914-z

**Published:** 2024-01-18

**Authors:** Xiaoye Wang, Changsheng Zhang, Tao Wang

**Affiliations:** 1 Northeastern University, Shenyang, 110819 P. R. China; 2 BMW Brilliance Automotive Ltd., Shenyang, 110143 P. R. China

**Keywords:** Electrical and electronic engineering, Mechanical engineering, Industry

## Abstract

In the automotive industry, machinery failures of the resistance spot welding (RSW) guns would interrupt the manufacturing lines and cause unplanned downtime, potentially resulting in a significant loss of production and reliability. Predicting the machinery failures of the RSW gun can provide more scientific strategies for predictive maintenance and decision-making. However, fault prediction of RSW guns has become increasingly challenging due to their complex behavior and data variability. In this paper, we created a benchmark dataset and proposed welding gun fault prediction benchmarks to aid in the development of machine learning approaches toward welding gun fault prediction. The dataset was collected at the Body-Shop (BS) of BMW Brilliance Automotive Ltd. from different components of hundreds of RSW guns to capture the patterns and trends before welding errors with historical data. Then we provide state-of-the-art machine learning (ML) benchmarks on time series forecasting methods in a welding gun fault prediction use case. This study will provide insights for time series forecasting while enabling ML researchers to contribute towards the fault prediction of the RSW guns.

## Background & Summary

Resistance spot welding (RSW) is one of the most popular welding procedures for low-carbon steel joining and is considered an ideal choice for assembly operation in many industry fields^1^. Especially for automotive manufacturing, over 90% of the steel body assembling work in the body-in-white process is completed by RSW^2^. Commonly the RSW is conducted by the servo-pneumatic RSW gun system, which is a mechatronic system with high speed and dynamic response and cooperates with the operation of the production line. Control errors of the servo-pneumatic RSW gun system (welding fault for short) can cause the production line to stop working and bring unplanned downtime. Since any unplanned downtime would result in productivity loss and extensive repair time, it is significant to utilize fault prediction technology to reduce downtime costs and improve welding guns’ working lives. In this context, fault prediction technology can take advantage of the unexploited lifetime potential of RWS guns by performing replacements before their failures occur.

The key capability of fault prediction is extracting useful knowledge from observed data, and a well understanding of the evolution through time before failures. Hence, great quantity and accurate data are of great importance for fault prediction to discover the potential working pattern of the welding gun, owing to relies extensively on past experience and observation. Conversely, RSW guns are highly nonlinear dynamical machinery systems with interesting physical phenomena over multiple time-dependent components. We believe that the breadth of the welding fault prediction problem can stimulate the development of new techniques in Machine Learning (ML). Recently, data-driven solutions for the welding domain have made considerable progress ranging from the weld nugget quality prediction^3^, welds diameter prediction^4^, process optimization^5^ and process control^6^. However, there is little existing research that focuses on the fault prediction of RSW guns, mostly because it is very expensive and time-consuming to collect defective welding data on the production line.

In order to fill the above research gap, we provide an open-source dataset along with welding gun fault prediction use cases and benchmarks for comparing welding gun fault prediction techniques. The dataset contains real-world multivariate time series data over 3 years from 80 RSW guns running at the production line of the body-shop of a leading car manufacturer. From the perspective of panel data, each time stamp contains 19 sensor parameters from different components of RSW gun labeled with one of the five kinds of machinery state. From the perspective of the time domain, each time series start at a normal working state and end up with a welding fault. The welding gun time series carries useful information about patterns and trends before welding gun faults. Moreover, we benchmark the performance of ML methods from the time series forecasting (TSF) domain for the RSW gun fault prediction task. It is our hope that this benchmark study will be useful for other researchers in the area of predictive maintenance in the automotive industry.

In summary, we provide an open-source dataset with fault prediction cases and benchmarks for the RSW gun from real word automobile manufacturing line. Our dataset provides future researchers with insights for fault prediction and machine health monitoring of the RSW gun while helping ML researchers interested in advancing the state-of-art in TSF. The dataset^7^ and benchmark codes^8^ are provided at Zenodo.

## Methods

In this section, we first describe the methods used to create the RSW dataset including data generation, data collection, data pre-processing, and data filtering. Then, we introduce the benchmark methods for the RSW gun fault prediction task.

### Data generation

In this subsection, we present the method for building the welding gun fault prediction dataset. We first give a detailed introduction to the servo-pneumatic RSW gun system, as the pre-knowledge of welding faults. We explain what welding faults are, how they are diagnosed, and why they are important. In the section collection, we introduce the physical properties measured for welding gun diagnosis and how to sample, transfer and store these data via the Internet of Things (IoT) platform. Next the technology adopted for data pre-processing and filtering is introduced in the pre-processing section and filtering section. Finally, the entire constructive process and the description of the welding gun fault prediction benchmark data set are shown at the end of this section.

#### Servo-pneumatic RSW gun system

The leading car manufacturer has been relying on the pinpoint accuracy and efficiency of servo-pneumatic welding guns for many years. The servo-pneumatic welding gun is a complex mechatronic system with high speed and dynamic response. The name servo comes from the servomechanism in control engineering. A servo controller is an automatic device that uses a closed-loop controller to correct the mechanism action. The name pneumatic is a driving mechanism commonly used in the industry and powered by compressed air or inert gases.

**Fig. 1:**
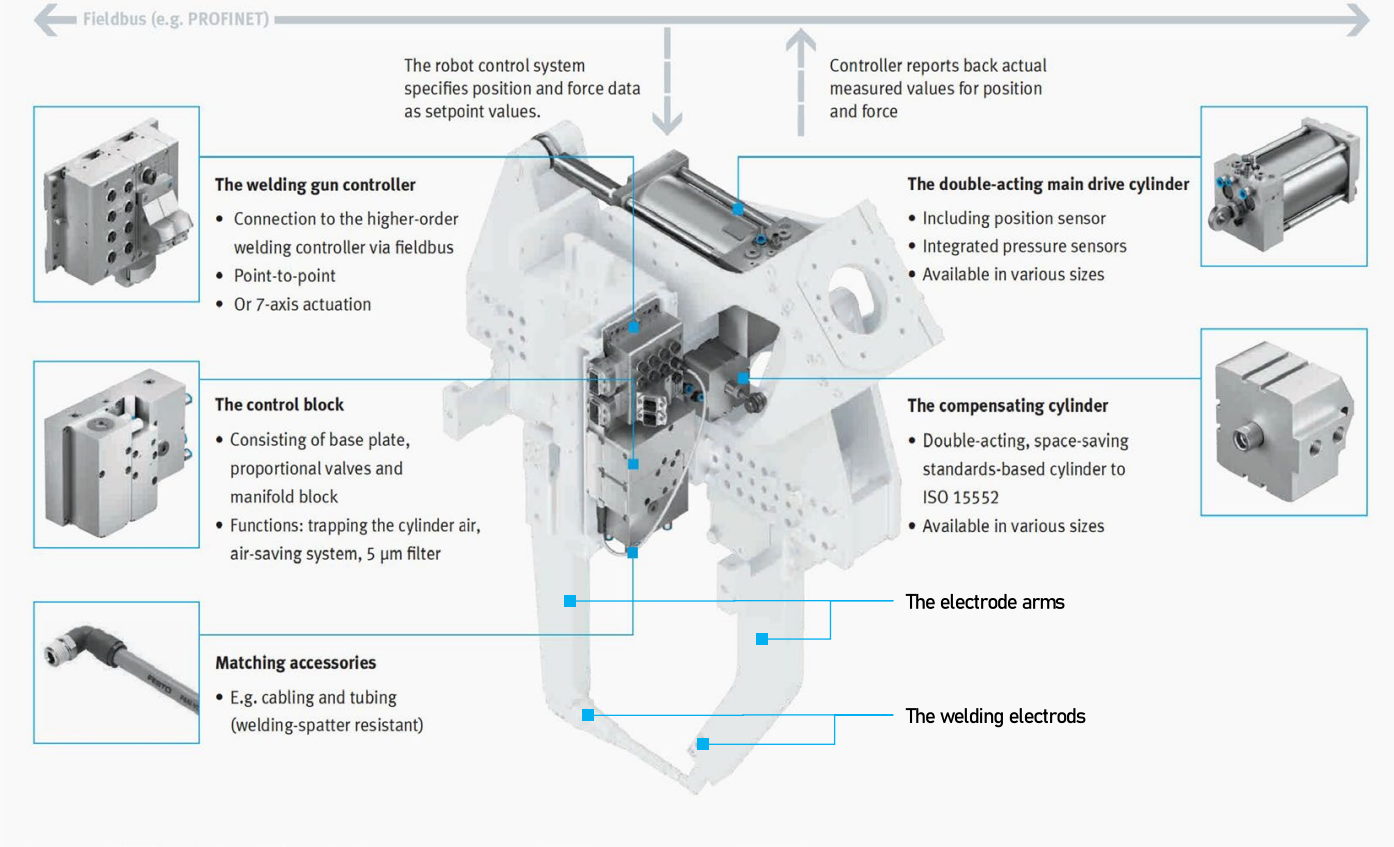
The essential components of the servo-pneumatic RSW gun system.

Figure 1 illustrates the essential components of a servo-pneumatic RSW gun system. The structure of the servo-pneumatic RSW gun system includes a controller, main drive cylinder, compensating cylinder, additional pneumatic circuit, and client-side software with a graphical interface. In terms of mechanical structure, the RSW gun is designed with two projecting electrode arms that are able to move relative to each other. One arm is connected to the main drive cylinder, enabling movement, while the other arm is fixed to the gun body, preventing any motion. Thus, the main drive cylinder causes the movable arm to move back and forth towards the fixed arm, causing the two welding electrodes to open and close in an alternating manner. When the electrode arms are open, they spread to the maximum distance between two electrodes to hold the welding sheets in place. When the electrode arms are in the closed position, they press the welding sheets together until the welding nuggets are completed.

In terms of electrical control, the controller connects to a higher-order welding controller with Fieldbus. The setpoints for position and pressure force data are provided to the controller. The controller converts the setpoint into electric control signals for the controller block. The controller block integrates various types of pneumatic control valve actuators. These valve actuators transform energy into mechanical motion. Mechanical motions are measured using the pressure sensor system and position encoder that are part of the main cylinder. These measured values are promptly sent back to the controller. The controller provides feedback of the actual measured values to the control system in order to correct the behavior of the welding gun. The components mentioned above form a closed control loop design for the information path. This design incorporates sensors, welding motion control algorithms, and pneumatic actuators. The servo controller uses negative feedback to detect errors and correct the motion of a mechanism, ensuring that the welding electrodes achieve the desired motion.

The failure of the RSW gun we are interested in is control errors of the servo-pneumatic RSW gun system. The control errors are triggered when a welding gun is working out of the control of the servo-pneumatic RSW gun system.

#### Welding fault in servo-pneumatic RSW gun system

The welding faults will occur when the servo-pneumatic RSW gun system is unable to control the welding gun movement. To be specific, if the bias between the measured value and setpoint exceeds a tolerable threshold, a motion control error will be diagnosed by the control system. The motion control error is the kind of welding fault focused on in this work. We will call it welding fault or welding failure in the rest of this paper. The occurrence of welding faults can happen at any stage of the production process. When a welding gun malfunctions, it requires maintenance before it can be used again. This can lead to unexpected downtime in the production line, which has a notable effect on productivity and dependability.

One way to reduce unexpected downtime is fault prediction. There are more than 20 welding gun fault types in the welding control system. Some of them are caused by man-made faults, some occur with a low possibility, and some are caused by the unhealthy condition of the welding gun. The unhealthy conditions could be arm cracking, arm deformation, gas leaking, and other unknown circumstances. These unhealthy conditions could have already existed for some time before the welding failure occurred. For example, when we repair a faulty gun, we see cracks in the electrode arm or sometimes a leaky cylinder. It is worth noting that these unhealthy conditions do not directly point to a unique and fixed welding gun failure. Conversely, the same welding gun failure can exhibit different kinds of unhealthy conditions when repaired. Therefore, there is not a one-to-one correspondence between the unhealthy conditions and the welding gun failure. Moreover, it is certain that unhealthy conditions always occur before the welding gun failure. Thereby, by analyzing the information before the welding gun fault, some welding gun faults are predictable.

We assess the frequency, downtime caused, and maintenance cost of each fault. Based on this evaluation, we will choose four fault types to predict in this study. These faults have similar features with high frequency, high risk, and high maintenance costs, which are detailed as follows:

- Counterbalance timeout error: The specified compensation pressure of the compensating cylinder is not reached for 1800ms;
- Electrode broke error: The electrode position is smaller than the zero position from the reference travel;
- Unwanted movement error: The main cylinder finishes its specified movement while the actual position value excursion the control value over 6.5% of the cylinder stroke;
- Drift error: The locked cylinder moves at a speed over 5 mm per minute instead of keeping still.

#### Collection

When collecting raw data on the welding process from production lines, the following three issues need to be concerned:

- What should be the appropriate data collection frequency?
  The sampling frequency of data collection should be high enough to ensure temporal dependence features are adequately carried. However, it should not be over-intensive in case of causing control signal congestion in the welding control system. Considering that unhealthy conditions take time to develop as discussed previously, millisecond sampling frequency is obviously too intensively. Therefore, according to the welding experience in practical production, we set the sampling frequency at 1 sample point per second.
- What type of features should be gathered?
  We gather the following five types of features:
  - Control parameters: Control parameters are setpoints used to correct the welding action. Higher-order control commands provide them as an ideal welding parameter and operating standard. Since the welding faults are cached by the error-sensing of the feedback mechanism in servo control, the control parameters provide positive feedback information in the control loop.
  - Cylinder parameters: Air cylinders are pneumatic motors that perform mechanical movement by means of compressed air. The movement of the cylinder is specified by setpoints from the controller. Since the welding faults are sensed when welding motion is incorrect, the measured cylinder parameters provide negative feedback information in the control loop. So far, both positive and negative feedback information complete the closed loop of servo-pneumatic control.
  - Operating status parameters: There are three electrode states (i.e., stationary, pre-contact, and post-contact) in the production line. In the stationary state, ‘two electrodes hold a long distance from each other and remain relatively stationary. In the pre-contact state, the two electrodes move close to each other but do not touch the steel sheet. In the post-contact state, the two electrodes touch and clamp the steel sheet. Different welding faults may occur in different electrode states. For instance, the drift error occurs only in the stationary state, the unwanted movement error occurs in both two of the pre-contact and post-contact states, and the electrode broke error can occur in all three states.
  - Electrode monitoring parameters: In the post-contact state, two electrodes continue to move toward each other after contacting the surface of steel sheets. Meanwhile, the two electrodes squeeze and clamp the steel sheet, and release the electric current. In this way, the surface of steel sheets is joined by heat generated from the resistive current. The above process is always monitored through electrode parameters to evaluate the welding process. Therefore, the electrode monitoring parameters are circular for welding gun motion control fault prediction.
  - Welding gun information: The working environment is an undetermined factor affecting the working situations of the welding gun. As such, customized information about the welding gun should be concerned, e.g., cumulative welding points of welding gun and working area of welding gun.

- How to collect these parameters?

The control parameters can be collected from the controller signal, including counterbalance pressure, electrode force, electrode position, velocity, and force build-up^9,10^. The cylinder parameters can be measured inside the main cylinder by sensors. The detailed sensor measurement solution is shown in Fig. 4. Two sets of sensors are included inside the main cylinder, one position sensor and two pressure sensors. The position sensor is integrated with a built-in measuring system, which measures the piston position relative to the length of the mechanical stroke. Two pressure sensors measure the pressure on each side of the piston. The operating status parameter, the electrode monitoring parameters, and information of the welding gun can be queried from the higher-order control system, the intelligent welding systems (IWS)^11,12^, including friction, maximum aperture, maximum electrode force, start friction, US2 status, offset value in the robot, time of data, error type, welding point count, position count, area, gun id, IP, robot name, station, and PLC status.

- How is the collected data transmitted and organized?

The fourth aspect is the data collection platform. As mentioned above, the servo-pneumatic RSW gun system has already processed a lot of control process data and compresses it to the user graphic interface on the client side so that it can be used as diagnostics. Unfortunately, the control system simply provides and displays data in real-time without saving them. To save the historical data, we built a data collection platform based on the IoT composed of welding controllers, the programmable logic controller (PLC), the IoT gateway, and the cloud database, as shown in Fig. 3. In the entire platform architecture, the welding controller is responsible for obtaining real-time welding data and controlling the welding motions. However, it lacks sufficient memory to process or store the welding parameters. Thus, the welding parameter data in real-time is sent to the PLC through input interfaces for further processing. The real-time data is stored in the memory of the PLC for sampling and processing at the specified frequency. Then, the processed data is transmitted to the IoT gateway through its communications interface. The IoT gateway enhances data transmission security by facilitating communication between the PLC and the cloud end. In the cloud environment, a stream data database is used to transform real-time data into time series data. This data is converted and stored as historical data, making it easy to query and analyze.

In summary, we collect welding data from the real-world body shop of car manufacturers since 2019. From all these data, We select 80 welding guns that work continuously for more than 7 days and finally end up with a welding error. No other exclusion criteria were applied.

#### Pre-processing

In general, raw data collected in real industrial environments is not directly usable for analysis. After data collection, we need to apply data pre-processing operations to fix the welding data imperfections: missing data and outliers.

The missing data is caused by sensor faults, IoT network failures, communication transmission latency, and other operational issues. Missing data leads to reductions in the amount of information it carries. Based on practical production experiences, welding gun data with a missing rate greater than 40 percent carries very little information. Moreover, missing data causes discontinuity in timestamps. Outlier data is generated by random events unrelated to the welding process, such as the cap dressing and cap changing.

For the above problems of raw data, we carry out the following data pre-processing operations according to the working characteristics of the welding gun^13^. We first resample the sensor signal with 1 sample per second to blank the missing data. Then we calculate the missing rate of each gun and abandon the data of whose missing rate is larger than 40 percent. Next, we find out the outliers by inspecting the welding control parameter. If the control parameter “sheet thickness” is larger than 6 it means the operation of the welding gun at that point is not welding. This kind of data is treated as outliers and also revalued as blank. In the end, we fill the blank data with the most recent data point which is correctly collected. This pre-processed data is used by the next filtering step, but we retain the missing data and outliers in the published dataset. The intention is to promote more robust methods for fault prediction with imperfections data from real-world industry applications.

#### Filtering

Once the welding parameter data is clean, we move to the final step of the methodology which is data filtering. This stage produces useful variables for the welding gun fault prediction task. We use the correlation coefficient to measure how strong a relationship is between each two welding parameters. First, we cut all the time series data into sub-time-series by using a sliding window, in which the width and step length are both equal to *l*. The *n*-th sub-time-series of the welding gun *g* is denoted as ${S}_{n}^{g}=\left\{s{(1)}_{n}^{g},\ldots ,s{(D)}_{n}^{g}\right\}$. Then we use the series correlation matrix (SCM) to calculate the correlation coefficient between each welding parameter on each sub-time-series. The SCM of the *n*-th sub-time-series of the welding gun g is denoted as:

1 $$SC{M}^{n}=\left[\begin{array}{ccc}{R}_{11}^{n} & \cdots  & {R}_{1D}^{n}\\ \vdots  & \ddots  & \vdots \\ {R}_{D1}^{n} & \cdots  & {R}_{DD}^{n}\end{array}\right]$$

in which ${R}_{i,j}^{n}$ is the Pearson correlation coefficient (PCC) between the *i*-th welding parameters ${S}_{i}^{n}$ and the *i*-th welding parameters ${S}_{j}^{n}$ range in [−1, 1] and calculated as:

2 $$\begin{array}{l}{R}_{ij}^{n}=SCP\left({S}_{i}^{n},{S}_{j}^{n}\right)\\ =\left\{\begin{array}{ll}\frac{\mathop{\sum }\limits_{t=t{\prime} }^{t{\prime} +l}\left(s{(t)}_{i}^{n}-\overline{{s}_{i}^{n}}\right)\left(s{(t)}_{j}^{n}-\overline{{s}_{j}^{n}}\right)}{\sqrt{\sum {\left(s{(t)}_{i}^{n}-\overline{{s}_{i}^{n}}\right)}^{2}}\sqrt{\sum {\left(s{(t)}_{j}^{n}-\overline{{s}_{j}^{n}}\right)}^{2}}} & i\ne j\\ 0 & i=j\end{array}\right.\\ \overline{{s}_{i}^{n}}=\frac{\mathop{\sum }\limits_{t=t{\prime} }^{t{\prime} +l}s{(t)}_{i}^{n}}{l}\\ \overline{{s}_{j}^{n}}=\frac{\mathop{\sum }\limits_{t=t{\prime} }^{t{\prime} +l}s{(t)}_{j}^{n}}{l}\end{array}$$

in which $s{(t)}_{i}^{n}$ is the value of sub-time-series ${S}_{n}^{g}$ at time *t*. $\overline{{s}_{i}^{n}}$ and $\overline{{s}_{j}^{n}}$ respectively are the mean of $s{(t)}_{i}^{n}$ and $s{(t)}_{j}^{n}$ on ${S}_{n}^{g}$. Finally, we calculate the entirety correlation coefficient by the mean of the correlation coefficient of the sub-time-series:

3 $$SCM=\left(\begin{array}{ccc}{R}_{11} & \cdots  & {R}_{1D}\\ \vdots  & \ddots  & \vdots \\ {R}_{D1} & \cdots  & {R}_{DD}\end{array}\right)$$

4 $${R}_{i,j}=\left\{\begin{array}{ll}\frac{\mathop{\sum }\limits_{g=1}^{G}\mathop{\sum }\limits_{n=1}^{N}{R}_{ij}^{(n,g)}}{N* G}, & i\ne j\\ 0, & i=j\end{array}\right.$$

In this step, 19 parameters are relevant, and in the next time series forecasting task, we will only use the relevant parameters of the target parameters for forecasting^7^.

**Fig. 2:**
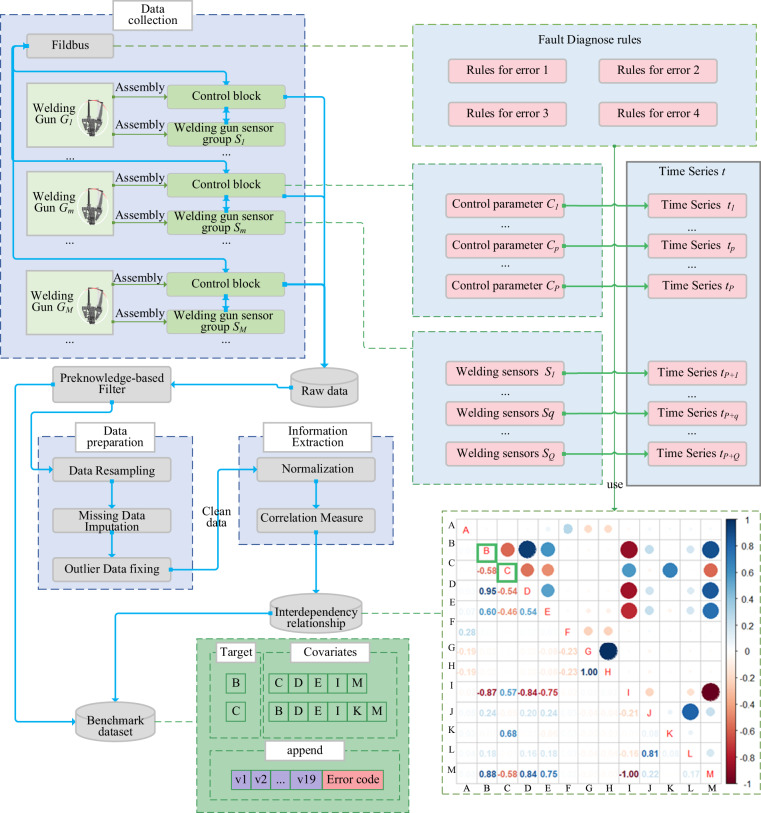
The constructive process and the description of the welding gun fault prediction benchmark data set.

So far, all aspects of the RSW database generation have been introduced. Figure 2 shows the complete generation process of the RSW database. First, we collect the data from the servo-pneumatic RSW gun system. We denote $G=\left\{{g}^{1},{g}^{2},\ldots ,{g}^{m},\ldots ,{g}^{M}\right\}$ as a set of welding guns, in which *g*^*m*^ is the *m*-th welding gun. Each welding gun *g*^*m*^ is sent a set of parameters ${C}^{m}=\left\{{c}_{1}^{m},{c}_{2}^{m},\ldots ,{c}_{p}^{m}\right\}$ from the IWS, in which ${c}_{p}^{m}(p=1,\ldots ,P)$ is the *p*-th control parameters of *g*^*m*^. Each welding gun *g*^*m*^ is equipped with a set of cylinder sensors ${S}^{m}=\left\{{s}_{1}^{m},{s}_{2}^{m},\ldots ,{s}_{q}^{m},\ldots ,{s}_{Q}^{m}\right\}$ to characterize quantitatively information about welding parameter, in which ${s}_{q}^{m}(q=1,\ldots Q)$ is the *p*-th sensor of *g*^*m*^. Then, we apply multiple pre-processing techniques including data resampling, data imputation, and data analysis.

**Fig. 3:**
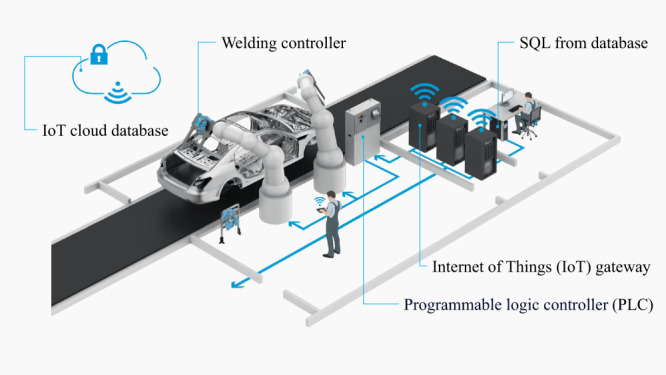
Collecting the welding gun data to the cloud via the IoT platform.

**Fig. 4:**
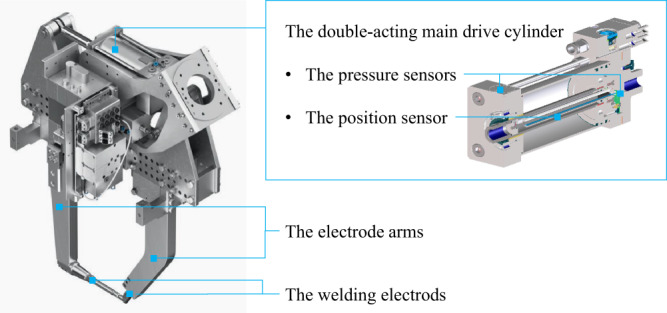
The internal structure and sensors of the main double-acting cylinder.

### Machine learning benchmarks

In this subsection, we introduce the goal of the welding gun fault prediction task and present the benchmark of the popular learning methods in terms of solving the welding gun fault prediction task.

#### Problem definition

ML-based approaches for welding gun fault prediction require the availability of historical welding data. Specifically, a sequence of welding data, named welding series, can be considered as a multivariate time series *X*, and each timestamp of *X* is a vector composed of all the collected welding parameters. Let the vector of welding series *X* at time *t* be *x*_*t*_ and welding fault happens at time *t* be *y*_*t*_. In welding gun fault prediction benchmark data set *y*: = {Counterbalance timeout error, Electrode broke error, Unwanted movement error, Drift error, Normal} as mentioned above. The goal of welding gun fault prediction is to learn a model ${\mathscr{F}}:({X}_{1:{t}_{0}-1})\to {y}_{T}$, that can map the past time series $[{x}_{1},...,{x}_{{t}_{0}-1}]\,:={X}_{1:{t}_{0}-1}$ to welding fault *y*_*T*_, where *t*_0_ denotes the time point from which the welding parameters are unknown. To prevent confusion, we avoid using the ambiguous terms “past” and “future” in the rest of the paper, instead, we refer to time ranges [1, *t*_0_ − 1] and [*t*_0_, *T*] as *conditioning range* and *prediction range*, respectively.

Welding parameters can be distinguished into two different kinds: target parameters and covariate parameters. The target parameters (i.e., electrode force and balance pressure) trigger the welding gun fault directly, and the covariate parameters trigger the welding gun fault indirectly by impacting the target parameters. A sequence of target parameters, named target series, can be considered as a multivariate time series *Z*, and each timestamp of *Z* is a vector composed of all the target parameters. Denoting the vector of target series at time *t* by *z*_*t*_, welding faults are detected in the servo-pneumatic RSW gun system by diagnosis rule ${\mathscr{H}}:({Z}_{1:t})\to {y}_{t}$, that can map target series $[{z}_{1},...,{z}_{t}]\,:={Z}_{1:t}$ to the welding fault *y*_*t*_. Practically, the rule for welding fault diagnosis are known by welding experts in real-world production. Based on this situation, ${\mathscr{H}}$ can be considered as a priori knowledge for solving the fault prediction problem. In this case, if the target series in the prediction range is diagnosed by ${\mathscr{H}}$, the welding fault can be predicted. In other words, the welding fault prediction task can be solved by accurately forecasting the target series in the prediction range.

Thus, we use a two-stage strategy to solve the welding gun fault prediction task. In the first stage, we learn a model ${\mathscr{G}}:({X}_{1:{t}_{0}-1})\to {Z}_{{t}_{0}:T}$, that can map ${X}_{1:{t}_{0}}$ to the prediction range of the target series $[{z}_{{t}_{0}},...,{z}_{T}]\,:=\,{Z}_{{t}_{0}:T}$. In the second stage, ${\mathscr{H}}$ map the target series ${Z}_{1:{t}_{0}-1}+{Z}_{{t}_{0}:T}\,:\,={Z}_{1:{t}_{T}}$ to *y*_*T*_. In this strategy, the core is modeling ${\mathscr{G}}$ by time series forecasting technology since ${\mathscr{H}}$ is the priori knowledge.

#### Benchmarking methods

For broad and representative method competition for the welding fault prediction task based on the RSW dataset, different time series forecasting methods are compared in this work. Methods that only support univariate time series, usually statistical methods, are not considered competitors in this case. So are the methods that don’t support past-observed covariates time series. Considering the welding series are multivariate and some components of the time series are forecast targets and the others are the covariates time series which don’t need to be forecast but are relevant to the target time series. Another category called Local Forecasting Model (LFM), usually classical machine learning methods, can learn a local model from an isolated time series, meaning that they are trained on the history of a single time series and forecasts the future of this time series. Since we want to consider the history of many series of different welding guns and predict the welding gun fault by a global forecaster, LFM is also not considered. Global Forecasting Model (GFM), usually a deep-learning method, can learn a global model from multiple time series data, meaning that they can be trained on multiple time series and forecast multiple time series in one go. These category forecasting methods are competitors in our case. The competitors can be grouped into (i) machine learning methods; (ii) deep learning methods. For each category, we consider a set of representative methods. The machine learning forecasting methods are briefly described below:

Regression forecasting models forecast the future values of a target series based on lagged values, including linear Regression^14^, bayesian ridge regression^15^, random forest regression^16^. The scikit-learn package in python can be used to fit these models to predict the target time series from lagged values. LightGBM^17^ is a gradient-boosting framework based on decision trees. It’s a free open-source framework developed by Microsoft. It uses two novel techniques: Gradient-based One Side Sampling and Exclusive Feature Bundling (EFB) which allow the algorithm to run with higher efficiency, smaller memory, and a high level of accuracy at the same time.

The competing methods from the field of deep learning are outlined in the following:

- Block Recurrent Neural Network Model (RNNs) is a sequential neural network model that uses an RNN encoder to encode fixed-length input chunks, and a fully connected network to produce fixed-length outputs^18–20^. It’s popular due to the broad applications in language modeling^21–23^ and machine translation^24,25^. Three variants of RNNs are commonly used instead, such as the LSTM^26^ and the GRU^27^.
- Neural Basis Expansion Analysis Time Series Forecasting (N-BEATS)^28^ is a type of neural network that outperformed all other methods in the M4 competition^29^. Being different from the RNNs model, N-BEATS implements a “pure” deep neural architecture. At first, it was the state-of-art univariate time series forecasting method, but nowadays implementation also supports multivariate series by flattening the model inputs to a 1-D series and reshaping the outputs to a tensor of appropriate dimensions.
- Temporal Convolutional Network Model (TCN)^30^ is a revolution for time series forecasting solutions, which provides a unified approach to capture both low levels of spatial-temporal information and high-level temporal information. It consists of causal 1D convolutional layers with the same input and output lengths.
- Temporal Fusion Transformers (TFT) is Google’s state-of-the-art attention-based Deep Neural Network for time series forecasting, optimized for great performance and interpretability^31^. In Google’s benchmarking research, TFT outperforms all benchmarks over a variety of datasets.

#### Benchmarking setup

We present the overall comparisons in this subsection. We build our time series using 32-bit data, and the tests are made on an Intel CPU Intel(R) Core(TM) i7-8700 CPU @ 3.20 GHz, with an NVIDIA GeForce GTX 1080 with 16 GB of RAM. All TimeSeries are pre-loaded in memory and given to the models as a list. To make a fair comparison, we normalize the numerical values to have zero mean and unit variance for stable training and imputed the missing value using KNN.

The target parameter is the electrode force for error E02 and the balance pressure for the other three errors. We use the other components of the welding time series as the past-observed covariates time series. All methods are run for 100 epochs with 32 batch sizes. The loss function used for training is MSE loss, which is a criterion that measures the mean squared error (squared L2 norm) between each element in the input and target. And we get the accuracy results from the iteration with the best score. The prediction methods have then been applied several times, allowing a better comparison of the performances.

In our experiments, each time series was divided into history and test, where the split depends on the lead time *N* of the welding gun fault prediction requirement, which in this study N = 60. We set the length of the input chuck equal to 20. Detailed parameters for every considered method are shown in Tables 1–7.

**Table 1:** Detailed parameters for Bayesian Ridge methods.

Parameter	Value
Stop the algorithm if *w* has converge	1*e*-3
Alpha 1	1*e*-6
Alpha 2	1*e*-6
Lambda 1	1*e*-6
Lambda 2	1*e*-6

**Table 2:** Detailed parameters for RF methods.

Parameter	Value
Lags	20
Past covariates leg	20
The number of estimators	100
The maximum depth	None

**Table 3:** Detailed parameters for N-BEATS method.

Parameter	Value
Input chunk length	20
The number of stacks	20
The number of blocks	30
The number of layers in each block of every stack	4
Layer widths	256
Expansion coefficients dimension	5
Trend polynomial degree	2
Activation function	ReLU
Random state	42

**Table 4:** Detailed parameters for LightGBM methods.

Parameter	Value
Lags	20
Random state	42

**Table 5:** Detailed parameters for RNNs methods.

Parameter	Value
Hidden size	64
Number of layers in the RNN module	2
Dropout	0.2
Random state	42

**Table 6:** Detailed parameters for TCN method.

Parameter	Value
The size of every kernel in a convolutional layer	3
The number of filters in a convolutional layer	3
The base of the exponent	2
Whether to use weight normalization	False
Dropout	0.2
Random state	42

**Table 7:** Detailed parameters for TFT method.

Parameter	Value
Encoder length	20
Decoder length	60
Hidden state size	512
Number of attention heads	4
Number of layers for Encoder and Decoder	1
Dropout	0.2
Random state	42

#### Applied performance measures

We compare the point forecast accuracy of the above methods on the welding gun fault prediction benchmark dataset. We consider four metrics based on a recent survey^32^ and to use common measures, the benchmark incorporates the Mean Absolute Error (MAE) error^33^, for two time series *y*^1^ and *y*^2^ of length *T*, it is computed as

5 $$\frac{1}{T}\mathop{\sum }\limits_{t=1}^{T}\left(\left|{y}_{t}^{1}-{y}_{t}^{2}\right|\right)$$

and the Mean Absolute Percentage Error (MAPE)^34^, given a time series of actual values *y*_*t*_ and a time series of predicted values ${\widehat{y}}_{t}$ both of length *T*, it is a percentage value computed as

6 $$100\cdot \frac{1}{T}\mathop{\sum }\limits_{t=1}^{T}\left|\frac{{y}_{t}-{\widehat{y}}_{t}}{{y}_{t}}\right|$$

And Mean Absolute Ranged Relative Error (MARRE), given a time series of actual values *y*_*t*_ and a time series of predicted values ${\widehat{y}}_{t}$ both of length *T*, is a percentage value computed as

7 $$100\cdot \frac{1}{T}\mathop{\sum }\limits_{t=1}^{T}\left|\frac{{y}_{t}-{\widehat{y}}_{t}}{{\rm{m}}{\rm{a}}{{\rm{x}}}_{t}{y}_{t}-{\rm{m}}{\rm{i}}{{\rm{n}}}_{t}{y}_{t}}\right|$$

And Mean Squared Error (MSE), for two time series *y*^1^ and *y*^2^ of length *T*, is computed as

8 $$\frac{1}{T}\mathop{\sum }\limits_{t=1}^{T}{\left({y}_{t}^{1}-{y}_{t}^{2}\right)}^{2}$$

## Data Records

**Fig. 5:**
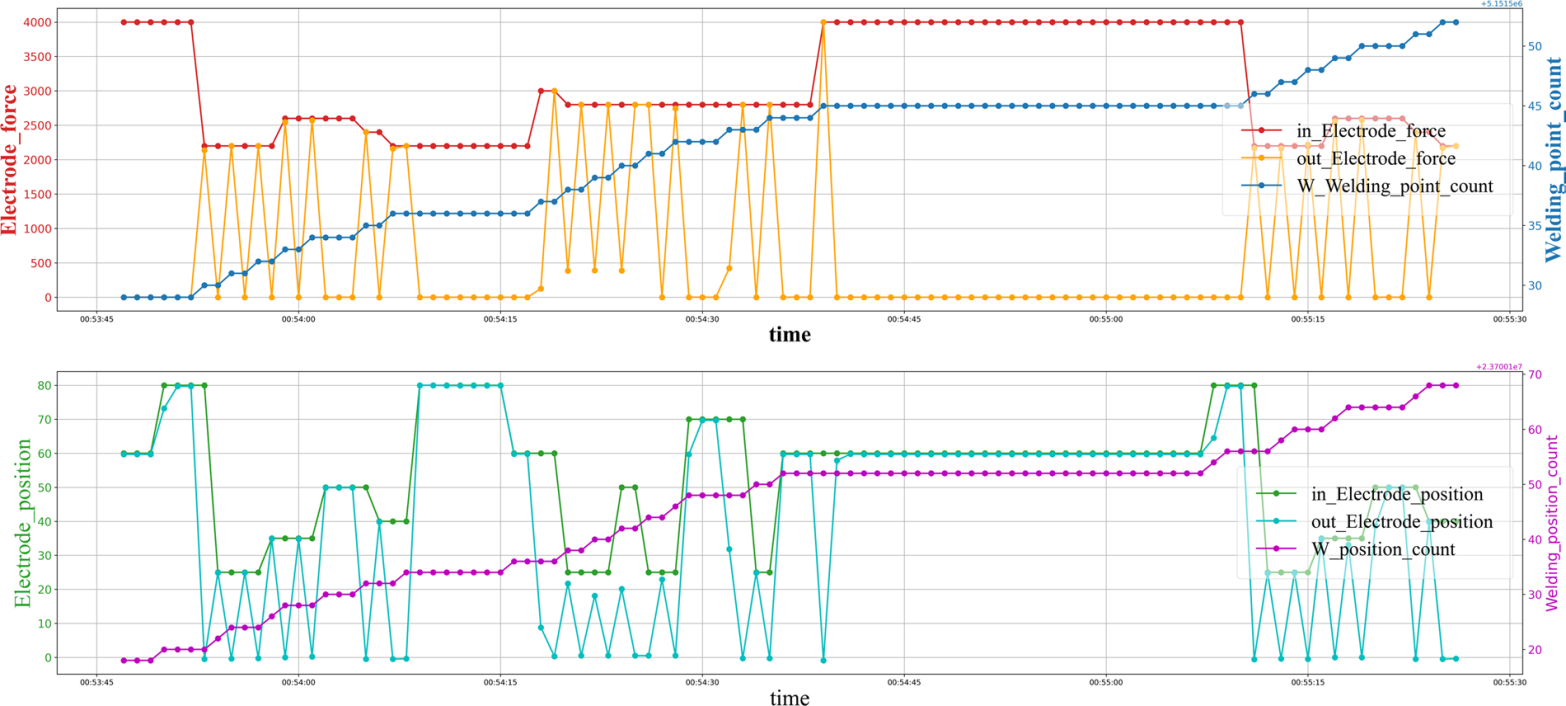
Visualization of RSW gun time series on some selected components. The “in Electrode position” and the “in Electrode force” are control parameters. The “out Electrode position” and the “out Electrode force” are welding parameters. The “W Welding point count” and “W position count” are counters of welding behavior.

The dataset is hosted on Zenodo^7^. The welding gun fault prediction benchmark data set has 72 multivariate time series (as shown in Fig. 5) in the training set and 8 time series in the testing set (see Table 8). Each time series length 604800 sampled at 1 Hz with missing values and has 20 dimensions (c1-c19 and the error code). There are five classes of error codes: E01, E02, E03, E04, and E00. E00 means no error (in the proportion of 1:1:1:1 both the training set and the test set). Table 9 gives an explanation of each error code. Table 10 gives a description of 20 dimensions. We retain the missing value and the outliers of the welding gun time series for the potential of imputation research in the future. We store the data in the CSV files by error code and gun id.

- File ERRORCODE_GUNNO.csv: for example, E02_16.csv is a CSV file containing sensor data of a gun ends up with error E02.test_2.csv is for the test without error code.
  - Field time: timestamp of the collection time.
  - Field c1: machinery parameter c1 of RSW.
  - Field c2: machinery parameter c2 of RSW.
  - ……
  - Field c19: machinery parameter c19 of RSW.
  - Field error: error code reported by the IWS in this timestamp.

**Table 8:** The data format of the welding gun fault prediction benchmark data set.

Train size	Test size	length	Number of class	Type
72	8	604800	5	numeric and enumeration

**Table 9:** The explanation of each error code.

Error code	Error name
E001	Counterbalance timeout
E002	Electrode broke
E003	Unwanted Movement
E004	Drift
E000	Normal state

**Table 10:** The description of variables in the multivariate time series.

Parameter	Discription
c1	Electrode cap offset
c2	Electrode force
c3	Electrode position
c4	Force build-up
c5	Balance pressure
c6	Friction
c7	Maximum aperture
c8	Maximum electrode force
c9	Mtart friction
c10	US2
c11	Welding point count
c12	Position count
c13	Setpoints of counterbalance pressure
c14	Setpoints of electrode force
c15	Setpoints of electrode position
c16	Setpoints of sheet thickness
c17	Setpoints of velocity
c18	Setpoints of force build-up
c19	Offset value in robot

## Technical Validation

### Exploratory analysis of dataset

Same with other real-world time series data, the characteristics of welding sensor data have a powerful influence on the prediction task. For example, missing values and outliers might induce bias in the forecasting model, patterns in the time series might contain important information to build the correct prediction model, and connectivity analysis focuses on identifying the interdependence relationships of the variables of the welding sensor data. All these characteristics should be considered when determining a suitable connectivity analysis scheme. In this section, we perform the exploratory analysis of welding data (as shown in Fig. 5): the missing value, the outliers, the pattern, and the interdependency relationships between sensor variables.

#### Irregularity and outliers

**Fig. 6:**
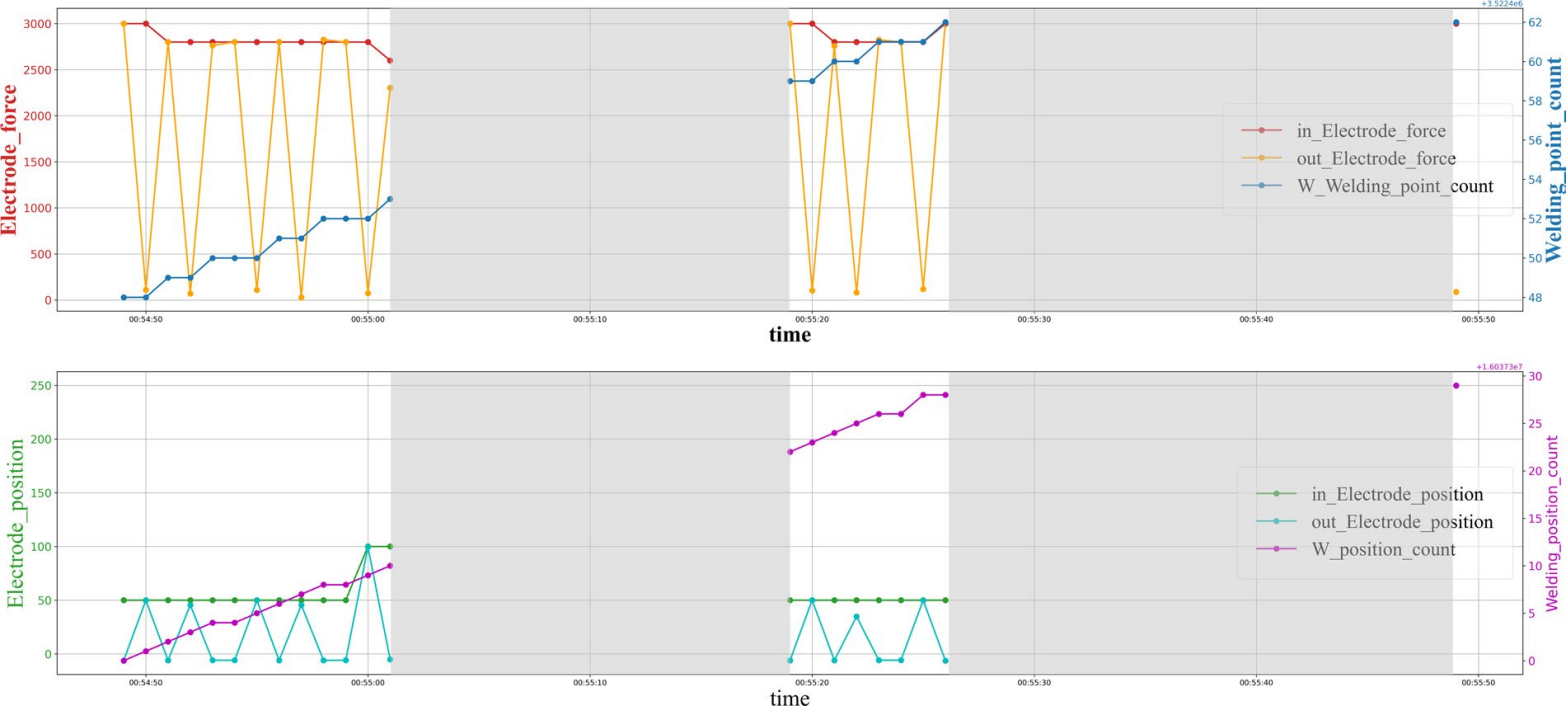
Visualization of the gaps on the RSW gun time series. The gaps are light gray colored backgrounds.

**Fig. 7:**
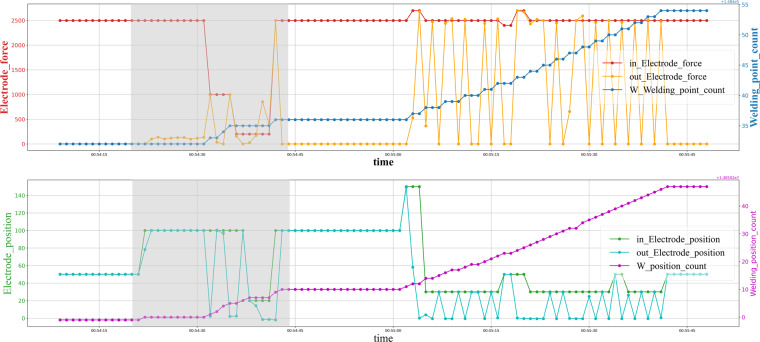
Visualization of the outliers on the RSW gun time series. The outliers are light gray colored backgrounds.

Control and sensor signals are sampled at a one-second frequency, but irregulars are caused due to signal failure, as shown in Fig. 6. Yet it is worth noting that the multiple components keep the consistency across dimensions. In most multivariate time series cases, irregular is a broad concept, “miss” specifically refers to the loss of a subset of components on a panel data but still has some left. But in our case, the reason for irregularity is the losing package of the sensor network, which means the whole panel data is missing at a particular time. Hence, we prefer using the word “block out” or “gap” instead of miss. Moreover, the block out also could be randomly or not randomly, and the data may lose a single panel or a long consistent period. On the other hand, random events unrelated to the welding process, such as the cap dressing and cap changing, cause outliers in the time series data of welding guns, as shown in Fig. 7.

#### Welding process

The welding process is used primarily for welding two or more metal sheets together by applying pressure and heat from an electric current to the weld area. During each welding process, the real electrode force parameter changes from zero to the control parameter rapidly and then changes back to zero after the welding nut is formed. The whole process persists for only hundreds of milliseconds, but the sampling frequency of the welding sensor data is 1 second. It leads to the unknowability of a single welding process, and the characterization of the time series data on the single welding process is unavailable for distinguishing normal and abnormal welding processes before a welding error happens. But on the upside, at least a split second of a welding process is sampled because it takes time for the welding gun to move from one welding nut to another. If we gather all the electrode force parameters with the same control parameter across long enough time series, we can still trace the rough process of parameter changing.

#### Welding behavior pattern

**Fig. 8:**
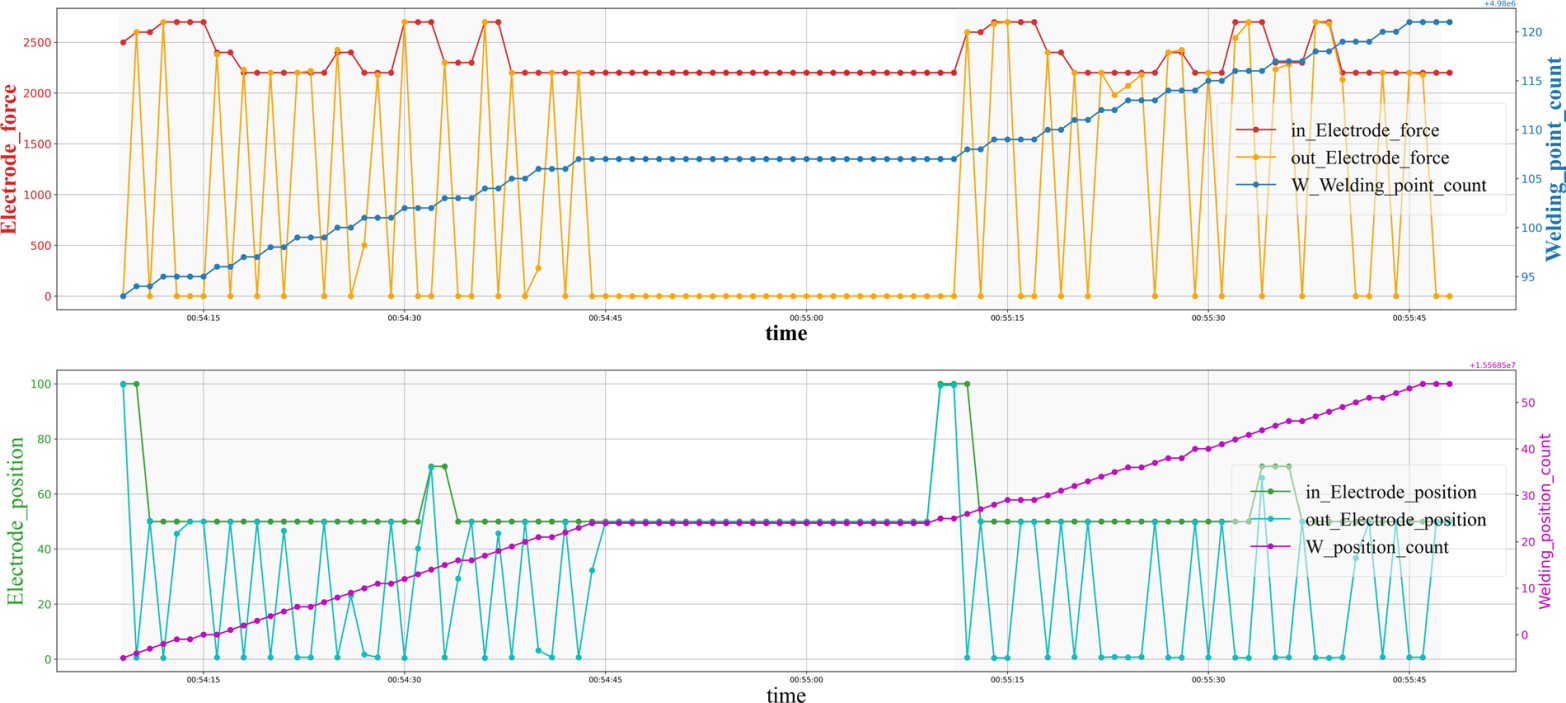
Visualization of the behavior pattern on the RSW gun time series. The patterns are light blue colored backgrounds.

Although a single welding process can’t be treated as the pattern of welding data because of the sampling frequency, the pattern still exists because of the particular behavior of the welding gun. There are thousands of weld nuts on a car body, and one welding gun is always in charge of the same series set of weld nuts., as shown in Fig. 8. If we see one sheet as a union, the process of generating a series of weld nuts on this sheet can be seen as a pattern, not precise but roughly consistent, and carry nice information for characterization.

### Time series forecasting method benchmark evaluation

#### Performance evaluation of MTS forecasting methods

**Fig. 9:**
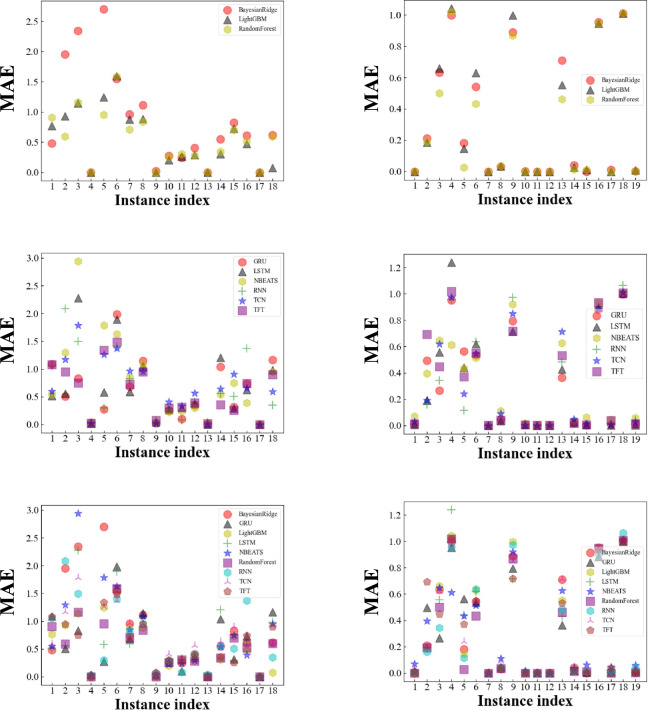
Scatter plots of the MAE obtained by MTSF algorithms. Randomly selected examples are used to show the forecasting results individually to explore the stability of the forecasting method. The results of the experiment are presented in two columns. In the left-hand column, the forecasting results for the target variable of electrode force; in the right-hand column, the forecasting results for the target parameter of balance pressure. The same examples are presented in each column. The examples shown between the two columns are different. Three types of presentation are used in each column, traditional machine learning class algorithms only, neural network class algorithms only, and all machine learning algorithms together.

**Fig. 10:**
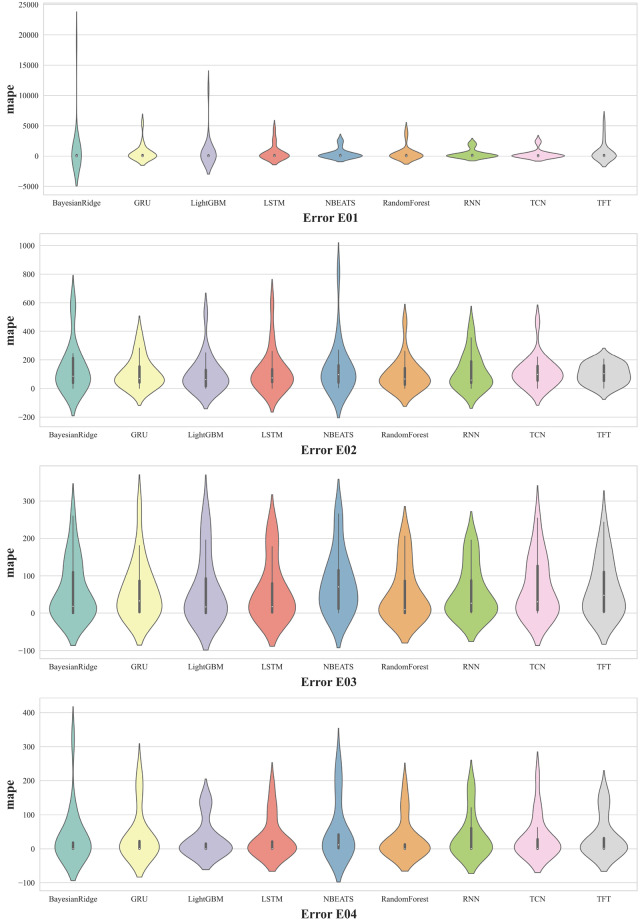
Violin plots of the MAPE obtained by the MTF method grouped in error class.

**Fig. 11:**
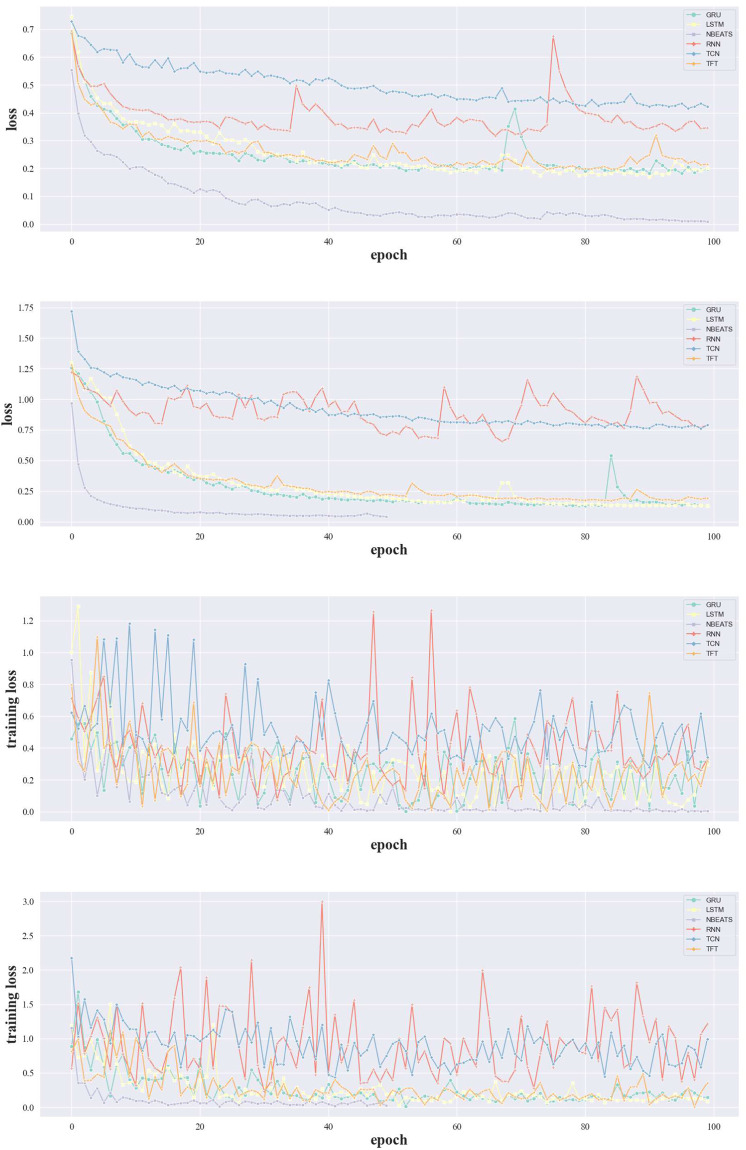
Scatter plots of the training process of the MTSF algorithms.

We investigate the performance of the forecasting methods. Bayesian Ridge and Linear Regression are used as baselines. Table 11 and Figs. 9–11 show the performance of the machine learning methods on all use cases. Each column in Table 11 shows a measure and each row shows a method. The best values (the lower, the better) are bold. The most accurate forecasting method based on MAE is TFT (0.2099) followed by random forest (0.2203). Concerning MAPE, the most accurate forecasting method is random forest (90.5216) followed by LightGB (148.2126) and the most accurate NN-based machine learning method is simple RNN (164.0906) followed by TFT (181.1341). Concerning MARRE, the most accurate forecasting method is TFT (5.33004) followed by random forest (5.5921). The most accurate forecasting method based on MSE is random forest (0.0958) followed by TFT (0.2099).

**Table 11:** The mean of the performance measure metrics obtained by MTSF algorithms.

algorithm	ame	mape	marre	mse
BayesianRidge	0.6047	174.0001	15.3520	0.7032
LinearRegression	0.5431	255.9767	13.7886	0.5156
RandomForest	0.2203	**90.5216**	5.5921	**0.0958**
NBEATS	0.3041	214.3311	7.7203	0.1671
LightGB	0.3174	148.2126	8.0568	0.2055
RNN	0.4008	164.0906	10.1751	0.2403
LSTM	0.8479	598.63796	21.52503	1.3111
GRU	0.6668	604.9237	16.9287	1.0064
TCNModel	0.9413	219.2912	23.8958	1.7227
TFT	**0.2099**	181.1341	**5.33004**	0.1047

After discussing the average performance of each method, we also investigate the stability of algorithm performance. We present the forecasting results of the many time series in groups. The groupings are based on forecasting algorithms and target parameters. The forecasting algorithms are divided into a traditional machine learning category, a deep learning category, and an all machine learning category. The target parameters are electrode force and balance pressure. The forecasting results will therefore be presented in six groups. In Fig. 9, we use six scatter plots to show the forecasting results for each of the six groups, measured in terms of MAE. In the left-hand column, the forecasting results for the target variable of electrode force; in the right-hand column, the forecasting results for the target parameter of balance pressure. On the electrode force prediction task, the RF has a stable performance the same as the mean MAE except for the first and the 18-th instance. But the TFT tends to lose accuracy on the 5-th instance, 11-th, and the 16-th instance while the RNN family got better performance than both RF and TFT. As for the balance pressure prediction task, the performance of some instances is nearly hard to distinguish because the task might be too easy in some instances. But on the other instance, every method got its best performance time. For example, RNN wins the score on the second instance. GRU leads the score on the third and 4-th instance. Then on the 9-th LSTM performed the best. It is hard to select the best-performed method among these instances.

Methods also perform differently on time series tending to different errors in the future. Figure 10 groups instance into 4 groups, each group representing the error to predict in the future. Error E01, E03, and E04 are diagnosed by the same sensor signal but their forecasting performance is quite different. On error E03 and error E04, all the method has good forecasting accuracy and RNN performs better than the others. But on error E01, BR and LightGBM produced large deviations than the others.

To demonstrate the overall training process, we also show the training curves on E01, E02, E03, and E04 based on the epoch in Fig. 11. NBEATS convergence very fast in all the cases but it didn’t perform well according to the earlier discussion. This infers the NBEATS tends to be easy to overfit on the welding gun fault prediction task than the other methods. Methods containing convolutional architecture convergence are more stable (e.g. TCN) than the sequence architecture (e.g. the RNN, LSTM, and GRU).

For all these measures, both classical and NN-based machine learning methods can be the most accurate machine learning method and in most cases, their performance is very close. The random forest method has the best performance among the other classical ML methods and the TFT perform better in the most case than the other machine learning methods. Additionally, the LightGB method and the RNN also have a good table performance under different measurements. But no method has absolute predominance in all kinds of instances.

#### Result of the welding gun fault prediction Task

In this section, we predict the welding gun parameter data in head 60 s using the best-performed MTS forecasting method in the benchmark section and then we map each forecasted horizon to a certain working state of the welding guns by the welding gun working state diagnoser. The welding gun working state diagnoser could be the heuristic rules which already exist or an analysis model based on the historical welding gun errors. It depends on the accuracy of forecasting results in the first step. From the results, one observes that the benchmarked method still has a big deviation in some particular timestamps, so a sensitive heuristic rule may lead to a false alarm. Hence, a window-based welding gun working state diagnoser is designed to evaluate the whole characteristic of the forecasted horizon based on the historical data of the welding gun time series.

We evaluate the performance of the proposed model using two measures, the average accuracy of the classifier and recall values for each class. These two evaluation metrics are specific to multi-class classification problems^35^. To be clear, we define the error gun as the positive class. By applying our welding gun fault prediction model directly to the predicted data, we obtained an average accuracy of 68.62%. This result can be considered dis-satisfactory when providing the welding gun predictive maintenance advice (See Table 12). However, a deep look at the recall values of each class reveals that the direct application of the algorithm favors the normal class by getting a recall value of 0.4431. This value suggests that nearly 56% of the normal welding gun working state was identified as an error by the algorithm. The respective recall values for error welding gun working state were 0.7431 suggesting that 74% welding error is predicted but with too many false alarms. The recall of each type of welding error is also present (See Table 12). The results are also far from satisfactory, it seems that the predicted welding sensor is not accurate enough to distinguish the error kind. These results imply that the MTSF algorithm plays an important role in the welding gun fault prediction task. Despite the obtained error recall, the direct application of the MTSF model fails to fulfill the requirements of the welding gun fault prediction task.

**Table 12:** Normal State or Error State Classification.

Evaluation Metrics	Results
Average Accuracy	68.62%
Recall for Normal	0.4431
Recall for error	0.7431
Recall for E01	0.3578
Recall for E02	0.1945
Recall for E03	0.2385
Recall for E04	0.1843

## Data Availability

Step-by-step guidance and the source code for machine learning benchmarks can be found on Zenodo^8^.
